# Evidence-Based Neonatal Unit Practices and Determinants of Postnatal Corticosteroid-Use in Preterm Births below 30 Weeks GA in Europe. A Population-Based Cohort Study

**DOI:** 10.1371/journal.pone.0170234

**Published:** 2017-01-23

**Authors:** Alexandra Nuytten, Hélène Behal, Alain Duhamel, Pierre-Henri Jarreau, Jan Mazela, David Milligan, Ludwig Gortner, Aurélie Piedvache, Jennifer Zeitlin, Patrick Truffert, On behalf of the EPICE (Effective Perinatal Intensive Care in Europe) Research Group

**Affiliations:** 1 Department of Neonatology, Jeanne de Flandre Hospital, Lille CHRU, Lille France; 2 Department of biostatistics, Univ. Lille, CHRU Lille, EA 2694—Lille, France; 3 Université Paris V René Descartes and Assistance Publique Hôpitaux de Paris, Hôpitaux Universitaire Paris Centre Site Cochin, Service de Médecine et Réanimation néonatales de Port-Royal, Paris, France; 4 Department of Neonatology, Poznan University of Medical Sciences, Poznan, Poland; 5 Newcastle University Newcastle upon Tyne, United Kingdom; 6 Children´s Hospital, University Hospital, University of Saarland, Homburg/Saar, Germany; 7 Inserm UMR 1153, Obstetrical, Perinatal and Pediatric Epidemiology Research Team (Epopé), Center for Epidemiology and Statistics Sorbonne Paris Cité, DHU Risks in pregnancy, Paris Descartes University, Paris, France; Centre Hospitalier Universitaire Vaudois, FRANCE

## Abstract

**Background:**

Postnatal corticosteroids (PNC) were widely used to treat and prevent bronchopulmonary dysplasia in preterm infants until studies showed increased risk of cerebral palsy and neurodevelopmental impairment. We aimed to describe PNC use in Europe and evaluate the determinants of their use, including neonatal characteristics and adherence to evidence-based practices in neonatal intensive care units (NICUs).

**Methods:**

3917/4096 (95,6%) infants born between 24 and 29 weeks gestational age in 19 regions of 11 European countries of the EPICE cohort we included. We examined neonatal characteristics associated with PNC use. The cohort was divided by tertiles of probability of PNC use determined by logistic regression analysis. We also evaluated the impact of the neonatal unit’s reported adherence to European recommendations for respiratory management and a stated policy of reduced PNC use.

**Results:**

PNC were prescribed for 545/3917 (13.9%) infants (regional range 3.1–49.4%) and for 29.7% of infants in the highest risk tertile (regional range 5.4–72.4%). After adjustment, independent predictors of PNC use were a low gestational age, small for gestational age, male sex, mechanical ventilation, use of non-steroidal anti-inflammatory drugs to treat persistent ductus arteriosus and region. A stated NICU policy reduced PNC use (odds ratio 0.29 [95% CI 0.17; 0.50]).

**Conclusion:**

PNC are frequently used in Europe, but with wide regional variation that was unexplained by neonatal characteristics. Even for infants at highest risk for PNC use, some regions only rarely prescribed PNC. A stated policy of reduced PNC use was associated with observed practice and is recommended.

## Introduction

Bronchopulmonary dysplasia (BPD) is a chronic lung disease frequently associated with very preterm birth [1,2] and defined as the need for oxygen at 36 weeks gestational age (GA) [3]. Inflammatory mechanisms in BPD have been described [4]. Therefore, postnatal corticosteroids (PNC) have been used to wean infants off ventilators [5]. In the 1990s, up to 25% of very preterm infants received dexamethasone [6].

Dexamethasone has been found to be effective for decreasing BPD, BPD or mortality, as a composite outcome, and facilitating extubation, but adverse effects include hyperglycemia, hypertension, gastrointestinal bleeding or perforation, and hypertrophic cardiomyopathy. Longer term consequences include a higher risk of cerebral palsy. [7–11]. The evidence for the use of other PNC is weak. There have been only a few trials of hydrocortisone to prevent BPD [12]. Only one study showed a reduction of BPD, but long term outcome wasn’t assessed [13]. Results from an observational study showing reduced white matter injury with betamethasone compared to dexamethasone in antenatal care [14] led to a preference for betamethasone as postnatal steroid therapy for BPD, but no trial results have been published for this indication. In 2002, the American Academy of Pediatrics and Canadian Paediatric Society published recommendations concerning PNC [15], revised in 2012 [16]: the routine use of PNC to prevent or treat BPD should be avoided; because of adverse effects, its use should be limited to exceptional clinical circumstances after parental information and PNC initiation should be delayed at least after 7 days of life.

After the publication of the AAP-recommendations, the use of PNC decreased [6,17,18]. However, the Models for OrganiSing Access to Intensive Care For Very Preterm Babies in Europe (MOSAIC) study showed that PNC was still in use in Europe in 2003, with rates from 4% to 35% among regions [19].

The Effective Perinatal Intensive Care in Europe (EPICE) study enrolled a population-based cohort of births before 32 weeks GA that occurred in 2011 and 2012 in 19 regions in 11 European countries. The study aimed to assess the use of evidence-based medicine for the care of very preterm infants. In this paper, we describe the rate of PNC use in participating EPICE regions and identify determinants of its use, including case-mix characteristics and neonatal intensive care unit (NICU) policies. Our hypothesis is that evidence-based policies can reduce PNC use.

## Methods

### Study design

The EPICE cohort is a geographically defined study of all very preterm stillbirths and live births from 22 +0 to 31 +6 weeks GA in 19 European regions. Data collection started between March and July 2011 and the inclusion period lasted 12 months, except in France, where it lasted 6 months.

### Study population

**Fig 1:**
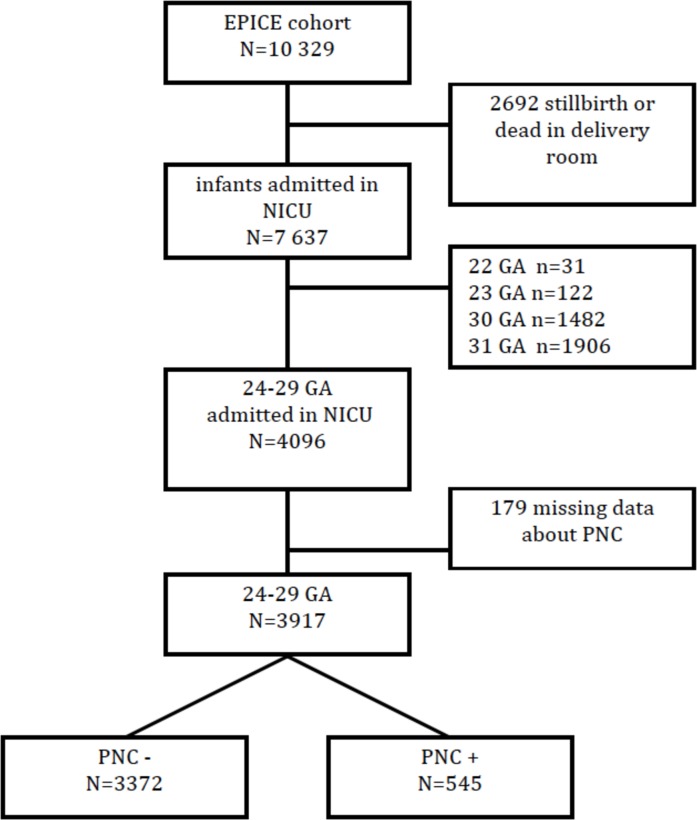
Flow chart of infants who met inclusion/exclusion criteria for the study. PNC, postnatal corticosteroids; NICU, neonatal intensive care unit; GA: gestational age.

For this study, all infants admitted to a neonatal unit were included. We excluded infants born before 24 weeks GA because of heterogeneous management of this subgroup in participating regions and infants born after 29+6 weeks GA because of reduced risk of BPD [20]. The flow chart is displayed in Fig 1.

### Data collection

Data on infants were collected using a standardised and pretested questionnaire from obstetrical and neonatal medical records. Data on the characteristics and practices of NICUs providing care for very preterm infants in participating regions were collected by use of a structured questionnaire sent to NICU administrative heads by mail or e-mail. Questions were formulated so that they could be answered in the same way by different staff members and several people within the NICU could discuss responses and complete the questionnaire. Data were collected on the structural characteristics of NICUs (level of specialization), their activity levels in 2011, policies, protocols and practices related to selected medical interventions, ethical decisions, decision-making processes and existence of healthcare quality monitoring systems.

NICUs with at least 10 very preterm admissions per year and their associated maternity units were included in the unit studies. These parameters were fixed before the study’s onset.

Ethics approval was obtained in each study region from regional and/or hospital ethics committees, as required by national legislation. The European study was also approved by the French Advisory Committee on Use of Health Data in Medical Research and the French National Commission for Data Protection and Liberties.

### Study outcome

The outcome in this study was the specific use of systemic PNC to treat BPD, including the type of steroid used, the initial dose, the age at initiation, and the length of treatment. When systemic steroids for this indication were given before 36 weeks GA, we considered they were used for infants with respiratory disease at high risk of BPD.

### Neonatal characteristics

GA was based on ultrasound measures and information on last menstrual period. Small for gestational age (SGA) was defined by a birthweight <10^th^ percentile by using references generated from all live births in the EPICE cohort. We distinguished babies with a birthweight between the 10^th^ and 25^th^ percentile as they have been found to be at higher risk of mortality and respiratory morbidity [21]. Birthweight, sex, type of pregnancy (singleton or multiple), mode of delivery, Apgar score at 5 min of life, surfactant therapy, mechanical ventilation, and use of non-steroidal anti-inflammatory drugs (NSAIDs) to treat persistent ductus arteriosus (PDA) were recorded. Antenatal steroids were scored as used if at least one dose had been given. Preterm premature rupture of membranes (PROM) was defined as a rupture of the membranes lasting longer than 12 hours before labor onset. Preeclampsia or eclampsia or Hemolysis, Elevated Liver enzyme Low Platelet (HELLP) syndrome was summarized as one variable.

### NICU policies on evidence-based practices

Using questions from the NICU questionnaire, we generated 2 variables to reflect each unit’s adherence to evidence-based practices. The first definition was based on the 2010 European guidelines for managing respiratory distress syndrome in very preterm infants [22]. We classified the NICU as having an evidence-based approach if the NICU reported that they administered surfactant to all newborns born before 26 weeks GA and to all newborns requiring intubation in the delivery room, as recommended. The second definition relied on declared policies concerning PNC prescription. We considered the NICU as having an evidence-based approach if it reported a “PNC restricted use policy”. The NICU was classified as evidence-based if it answered “no (or exceptional circumstances)” to both questions “In the unit, are systemic postnatal corticosteroids given to infants less than 32 weeks GA to prevent BPD” or “to treat BPD”. We analysed the effect of both approaches on PNC use separately.

### Statistical analysis

We used descriptive statistics to portray PNC prescription by region. We analysed the association between perinatal characteristics and PNC prescription by Student *t* or Mann-Whitney U test for quantitative variables and chi-square or Fisher exact test for qualitative variables. Variables with p < 0.2 on univariate analysis were included in a stepwise logistic regression analysis. The selected variables were consistent with the literature as risk factors for BPD [1,2]. For each infant, we then estimated the probability of receiving PNC by the logistic regression model, stratifying the sample by tertiles of probability of receiving PNC, as a risk profile, and selected the infants in the highest tertile (highest risk profile) for analysing PNC prescription. This subgroup included infants with the most severe respiratory condition. Finally, we analyzed the association between reported NICU policy for evidence-based practices and PNC prescription with a two-level hierarchical linear mixed model, with patients as the first level and the NICU as the second level. This last analysis involved the whole population and infants in the highest probability tertile. Results are expressed as crude and adjusted odd ratios (ORs) with 95% confidence intervals (95% CIs). All analyses were done using SAS 9.3 (SAS Inst. Inc., Cary, NC, USA).

## Results

### Use of PNC and variation across regions

Among newborns fulfilling inclusion criteria, 179/4096, or 4.3% had missing data on PNC prescription. The characteristics of the final sample retained for the analysis (3917 newborns) are described in Table 1. Infants with missing data had lower gestational ages (mean: 26.7 versus 27.1) and more often SGA (19% versus 9%) (p<0.05) (data not shown).

**Table 1:** Perinatal population characteristics.

Characteristics	Infants 24–29 weeks’ GA admitted to an NICU n = 3917	
Gestational age (weeks). mean (SD)	27.1 (1.6)	
Birth weight (g). mean (SD)	1105 (278)	
Gestational age		
24–25 weeks	748	(19.1)
26–27 weeks	1276	(32.6)
28–29 weeks	1896	(48.4)
Male gender	2139	(54.6)
Multiples	1173	(29.9)
Birth weight percentile		
SGA <10^th^	363	(9.3)
10-25^th^	614	(15.7)
>25^th^	2940	(75.1)
Preeclampsia/eclampsia/HELLP	558/3871	(14.4)
PROM	981/3859	(25.4)
Caesarean section	2551/3892	(65.5)
Inborn	3456/3911	(88.4)
Apgar <7 at 5 min	837/3644	(23.0)
Antenatal steroids	3465/3884	(89.2)
Surfactant	2983/3910	(76.3)
Mechanical ventilation	3030/3907	(77.6)
NSAIDs to treat PDA	1110/3899	(28.5)
PNC	545	(13.9)

Data are no. (%) unless indicated and for 3917 infants unless indicated.

GA, gestational age; HELLP: Hemolysis Elevated Liver enzyme Low Platelet NICU, neonatal intensive care unit; PNC, postnatal steroids; SGA, small for gestational age; PROM, premature rupture of membrane; NSAIDs, non-steroidal anti-inflammatory drugs; PDA, persistant ductus arteriosus

**Fig 2:**
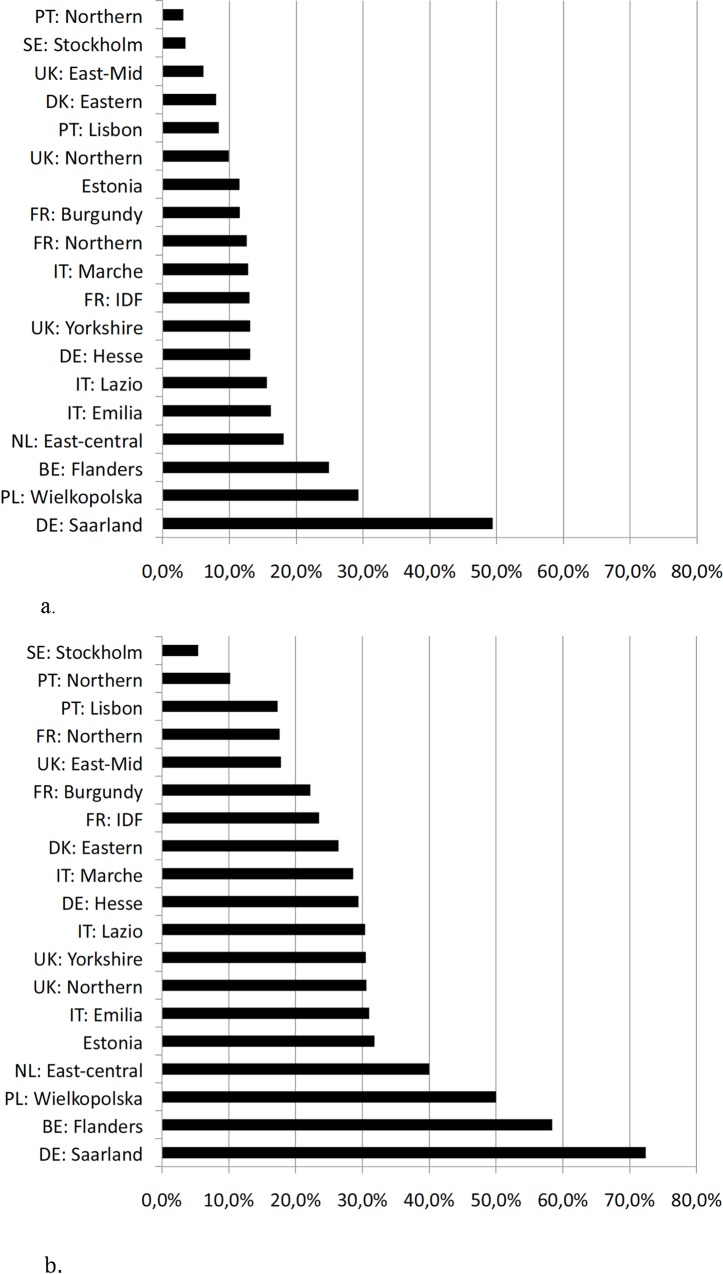
a) Rate of Postnatal Corticosteroid (PNC) use by region. b) Rate of PNC use by region for infants in the highest risk tertile. PT: Portugal, PL: Poland, UK: United Kingdom, SE: Sweden, DK: Denmark, FR: France, IDF: Ile de France, DE: Germany, IT: Italy, NL: The Netherlands, BE: Belgium,

**Fig 3:**
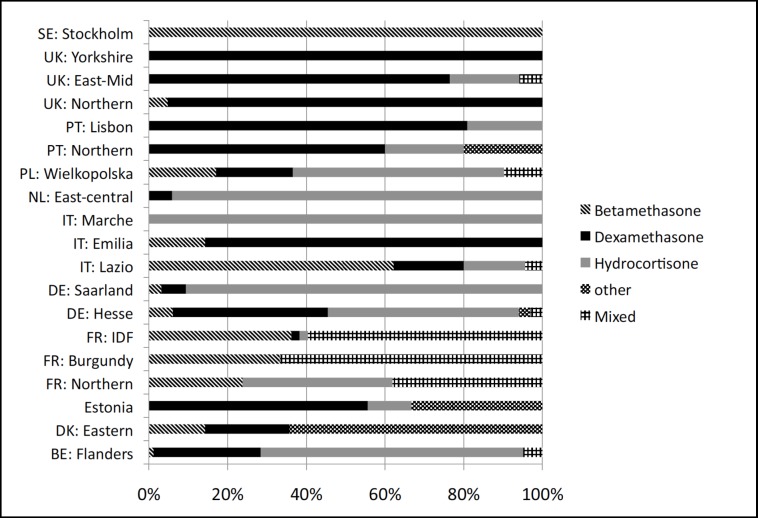
Type of corticosteroids used to treat bronchopulmonary dysplasia (BPD), by region. PT: Portugal, PL: Poland, UK: United Kingdom, SE: Sweden, DK: Denmark, FR: France, IDF: Ile de France, DE: Germany, IT: Italy, NL: The Netherlands, BE: Belgium,

In total, 545/3917 (13,9%) infants between 24 and 29 weeks GA received systemic PNC to treat BPD (Fig 1). Rates varied significantly among regions, from 3.1% (5/162 in northern Portugal) to 49.4% (39/79 in Saarland, Germany) (p<0.001) (Fig 2A). Dexamethasone, hydrocortisone and betamethasone were used in 195/545 (35,8%), 184/545 (33,8%) and 76/545(13,9%) of cases, respectively, and treatment was mixed for 59/545 (10,8%) of newborns (Table 2). Twenty six percent of infants (N = 50/195) received dexamethasone within the first 2 weeks of life. Twenty eight percent of infants received dexamethasone for more than 20 days (40 of 143 infants receiving dexamethasone, with data on treatment duration available). The type of drug used within regions varied significantly (p<0.001) (Fig 3).

**Table 2:** PNC use for infants at 24–29 GA (N = 545).

	Dexamethasone (35,8%)	Hydrocortisone (33,8%)	Betamethasone (13,9%)
Age at initiation (d)	24 (15–37)	12 (6–20)	21 (15–29)
Initial dose (mg/kg/d)	0.2 (0.15–0.5)	4.0 (1.7–5)	0.2 (0.1–0.3)
Treatment duration (d)	10 (6–20)	21 (11–29)	7 (4–12)
GA at birth (weeks) mean (sd)	25.6 (1.4)	26.0 (1.6)	26.5 (1.6)

Data are median (Q1-Q3) unless indicated.

### Neonatal determinants of PNC use

Low GA, SGA, male sex, mechanical ventilation and NSAIDs to treat PDA were significantly associated with PNC use after multivariate analysis (Table 3). Apgar score < 7 and surfactant treatment were no longer significant after adjustment.

**Table 3:** Use of PNC by neonatal characteristics.

	PNC		P value	crude		Adj	
	n/N	%		OR	95% CI	OR*	95% CI
GA							
24–25	242/748	32.4	<0.0001	8.7	6.7–11.2	5.6	**4.1–7.5**
26–27	204/1276	16.0	<0.0001	3.4	2.7–4.4	2.5	**1.9–3.3**
28–29	99/1893	5.2		-	-	-	**-**
Birthweight percentile							
<10th	79/363	21.8		2.0	1.5–2.6	2.1	**1.5–3.0**
10-25th	110/614	17.9		1.6	1.3–2.0	1.4	**1.1–1.9**
>25th	356/2940	12.1	<0.0001	-	-	-	**-**
Sex							
Female	210/1778	11.8	0.001	-	-	-	**-**
Male	335/2139	15.7		1.4	1.2–1.7	1.4	**1.1–1.8**
Multiple pregnancy							
Singleton	392/2744	14.3	0.30	-	-	-	-
Multiple	153/1173	13.0		0.9	0.7–1.1	0.9	0.7–1.2
PROM							
No	413/2878	14.4	0.11	-	-	-	-
Yes	121/981	12.3		0.8	0.7–1.0	0.9	0.7–1.1
Preeclampsia/eclampsia/HELLP							
No	448/3313	13.5	0.16	-	-	-	-
Yes	88/558	15.8		1.2	0.9–1.5	1.1	0.8–1.6
Caesarean							
No	208/1341	15.5	0.03	-	-	-	-
Yes	332/2551	13.0		0.8	0.7–1.0	1.1	0.9–1.4
Inborn							
No	68/455	14.9		1.10	0.8–1.4	0.7	0.5–1.0
Yes	476/3456	13.8	0.5	-	-	-	
Apgar score <7							
No	343/2807	12.2	<0.0001	-	-	-	-
Yes	150/837	17.9		1.6	1.3–1.9	0.9	0.7–1.2
Treatments							
Antenatal steroids							
No	62/419	14.8	0.62	-	-	-	-
Yes	482/3465	13.9		0.9	0.70–1.2	1.1	0.8–1.5
Surfactant							
No	36/927	3.9	<0.0001	-	-	-	-
Yes	509/2983	17.1		5.1	3.6–7.2	1.3	0.8–1.9
Mechanical ventilation							
No	10/877	1.1	<0.0001	-	-	-	-
Yes	534/3030	17.6		18.5	9.9–34.8	7.7	**3.8–15.8**
NSAIDs to treat PDA							
No	273/2789	9.8	<0.0001	-	-	-	**-**
Yes	271/1110	24.4		3.0	2.5–3.6	1.8	**1.5–2.3**

* adjusted on gestational age

SGA, sex, PROM, preeclampsia/eclampsia/HELLP:, caesarean section, apgar score, surfactant, mechanical ventilation, NSAIDs to treat PDA

OR: odds ratio; 95% CI: 95% confidence interval. n: number receiving PNC N: total in group

### Regional variation after case-mix adjustment

We used the five neonatal factors significantly associated with PNC use to calculate the probability of each infant receiving PNC. This probability could not be calculated for 28 infants because of missing data. We carried out subgroup analysis on infants with the most severe disease, defined as the tertile with highest probability of receiving PNC. The predicted probability of receiving PNC ranged from 17% to 66% in this group. In this subgroup, 392/1320 (29,7%) of infants received PNC to treat BPD and this varied significantly among regions, from 5.4% (2/37 infants) in Stockholm, Sweden to 72.4% (21/29 infants) in Saarland, Germany (p<0.001) (Fig 2B).

A total of 266 NICUs were located in the study regions in 2011, and 135 fulfilled the inclusion criteria. Of these, 134/135 (99,2%) provided completed questionnaires. In our sample, 69/3917 (1.8%) infants were excluded from this analyses because they were hospitalized in neonatal units that did not fulfill the inclusion criteria or did not complete the questionnaire. Twelve percent of neonatal units reported adherence to the European Association of Perinatal Medicine guidelines about surfactant use. Thirty six percent reported a PNC restricted use policy. We found no association between reported adherence to the European Association of Perinatal Medicine guidelines (21) and PNC use; however, infants hospitalized in units that declared having a policy concerning PNC restricted use were significantly less likely to receive PNC (Table 4).

**Table 4:** Association of NICU practices and PNC use.

Practice	No. of NICUs		No. Of infants		Infants at 24–29 weeks’ GA			Infants in the highest risk tertile		
	n/N	%	n/N	%	Adj OR*	95% CI	P value	Adj OR*	95% CI	P value
European Association of Perinatal Medicine recommendations	16/132	12	369/3719	10	0.91	0.4–2.1	0.83	0.82	0.3–2	0.65
NICU PNC restricted use policy	47/132	36	1069/3706	29	0.29	0.2–0.5	<0.001	0.29	0.2–0.5	<0.001

* adjusted on gestational age

SGA, sex, mechanical ventilation, NSAIDs to treat PDA

OR: odds ratio; 95% CI: 95% confidence interval. n: number using NICU Policy N: total in group

## Discussion

Our study showed that PNC are still used in Europe, with wide variations across regions. The main neonatal characteristics associated with PNC use were low GA, SGA, male sex, receiving mechanical ventilation, and PDA treatment with NSAIDs. However, these characteristics did not explain the wide variation in PNC prescription between regions, which persisted for infants with the most severe respiratory condition (highest risk tertile). NICUs with a PNC restricted use policy showed significantly lower PNC use.

For a quarter of infants who received dexamethasone, treatment was initiated in the first 2 weeks of life, and for another quarter it lasted for more than 20 days. Based on meta-analysis results and the latest recommendations, this use could be considered inappropriate [23,24]. Both hydrocortisone and betamethasone were commonly used to treat BPD and this could also be considered inappropriate as there was no evidence from trials at the time of the EPICE study suggesting that either is effective for BPD reduction. Since then one randomised controlled trial showed that hydrocortisone was effective for reducing BPD [13]. However there was no long term assessment.

Infants with severe respiratory conditions, dependant on mechanical ventilation after the first week of life, may benefit from PNC concerning BPD. However, our analyses by risk tertiles found that some low risk infants received PNC. Moreover, for infants in the highest-risk tertile, those with the most severe disease, some regions had low rates of PNC use showing that the variability in PNC use could not be explained solely by neonatal case mix and also that some regions used alternative strategies for managing severe respiratory complications.

We then considered evidence-based practices as a potential determinant of PNC use. Evidence-based medicine is the judicious use of current best evidence for making decisions about the care of individual patients [25]. The best evidence is from randomised controlled trials and meta-analyses. However, there is no clear indicator to measure use of evidence-based practices in neonatal medicine. We therefore developed two indicators. The first used the 2010 European Association of Perinatal Medicine (EAPM) guidelines [22] which relied on evidence published up to 2009 and which was released before EPICE, conducted in 2011/2012. In 2010, further studies showed less intubation and shorter mechanical ventilation for infants who received early continuous positive airway pressure [26,27]. Therefore, systematic surfactant became irrelevant, and was no longer recommended in the 2013 EAPM guidelines [24]. In 2011, as infants were included in the EPICE cohort, effective respiratory management evidence was changing. The second indicator was a stated policy of restricted PNC use. Evidence of PNC side effects is strong [8,28] and we considered a reported policy of restricted PNC use as an evidence-based practice [8,15,29]. Our definition selects NICUs with a real will to restrict PNC to exceptional circumstances. According to the current recommandations, dexamethasone should be restricted to infants under mechanical ventilation [9,30].

Our result that a lower probability of receiving PNC after consideration of case-mix was related to the existence of a unit policy of restricted use of PNC is consistent with Kaemps et al., an interventional study that showed that implementing local recommendations for respiratory management practices reduced dexamethasone use to treat BPD [31]. Our findings show an impact of explicit policies on real clinical practice [32] and support the development of clear unit policies for the use of evidence-based interventions or treatments.

While an explicit restricted use policy was a significant determinant of clinical practice, questions remain about the large practice variations observed in our study. This could reflect different interpretations of the evidence, in particular for infants at highest risk of BPD.

Doyle et al updated meta regression reported that in units with high rates of BPD, some children under assisted ventilation might have an increased chance of survival without CP [33]. However these results require confirmation at the individual level which would be possible in a randomized trial of infants at high predicted risk of BPD, as suggested by the authors.

The strengths of our study include its prospective population-based cohort design with few missing data. Data for the type of steroids, initiation date and dose and length of treatment were available. One limitation of our study is that our approach of evidence-based practices relied on a questionnaire and not on individual data. However, the declared PNC restricted use policy was associated with significantly reduced PNC use. Furthermore, despite a low missing data rate, newborns with missing data on PNC use were more immature and had lower birthweight than infants without missing data, which could have implied a selection bias and underestimated the PNC prescription rates. The next step will be to assess if low PNC use is associated with different mortality or survival without BPD rates among EPICE NICUs.

## Conclusion

European practices in PNC use for BPD are heterogeneous. Neonatal factors associated with PNC prescription reflected in part disease severity but did not explain the variation in PNC use. Our results suggest that a stated policy of restrictive PNC use could reduce non-evidence–based practices for individual patients. Other potential determinants such as knowledge concerning adverse effects, use of preventive respiratory strategies and saturation targets should be considered.

## Abbreviations

BPD: bronchopulmonary dysplasia
GA: gestational age
HELLP: Hemolysis, Elevated Liver enzyme Low Platelet
NICU: neonatal intensive car unit
NSAID: nonsteroidal anti-inflammatory drugs
PDA: persistent ductus arteriosus
PNC: postnatal corticosteroids
PROM: premature rupture of membrane
SGA: small for gestational age
WG: weeks’ gestation
