# Correction: Evidence-Based Neonatal Unit Practices and Determinants of Postnatal Corticosteroid-Use in Preterm Births below 30 Weeks GA in Europe. A Population-Based Cohort Study

**DOI:** 10.1371/journal.pone.0172408

**Published:** 2017-02-13

**Authors:** Alexandra Nuytten, Hélène Behal, Alain Duhamel, Pierre-Henri Jarreau, Jan Mazela, David Milligan, Ludwig Gortner, Aurélie Piedvache, Jennifer Zeitlin, Patrick Truffert, On behalf of the EPICE (Effective Perinatal Intensive Care in Europe) Research Group

The following information is missing from the Funding section: Funding for this open access publication was provided by the FP7 Post-Grant Open Access Pilot.
